# A Mandibular Metastasis as the Initial Presentation of Hepatoid Adenocarcinoma of the Lung: A Case Report

**DOI:** 10.7759/cureus.101740

**Published:** 2026-01-17

**Authors:** Georgios Kapes, Xheni B Merizaj, Ioannis Gakidis, Christos Chantziantoniou, Petros Michos

**Affiliations:** 1 Thoracic Surgery, General Hospital of Athens, KAT, Athens, GRC

**Keywords:** alpha fetoprotein, case report, hepatoid adenocarcinoma, lung, mandibular metastasis

## Abstract

Hepatoid adenocarcinoma of the lung is a rare primary pulmonary malignancy that exhibits morphological traits resembling those seen in hepatocellular carcinoma and is associated with high alpha-fetoprotein expression. It follows a very aggressive course and is often diagnosed in advanced oncologic stages. We present our experience with this malignancy and report on a case with mandibular metastasis, an uncommon metastatic site.

The patient, a 64-year-old male smoker with an estimated 50-pack-year history, was evaluated for a solid lesion in the left upper lobe, accompanied by hypertrophic pulmonary osteoarthropathy. He had undergone right partial gnathectomy for a symptomatic metastasis to the right submandibular region with pathology indicating hepatoid adenocarcinoma. Imaging revealed a 35×33 mm lesion in the left lung without nodal metastasis. Surgical management included a left upper lobe lobectomy and associated mediastinal lymphadenectomy, and the final diagnosis of primary hepatoid adenocarcinoma of the lung with neuroendocrine differentiation was confirmed. Despite chemotherapy, the patient developed bony metastases and succumbed four months post-surgery.

The above case highlights the aggressive nature of hepatoid adenocarcinoma, underscoring the importance of early diagnosis and the challenges posed by its metastatic spread. To our knowledge, this is the first reported instance of HAL presenting with mandibular metastasis as the initial manifestation, thereby expanding the known spectrum of metastatic sites.

## Introduction

Hepatoid adenocarcinoma (HAC) is an uncommon extrahepatic malignancy noted for its aggressive behavior and morphological similarity to hepatocellular carcinoma (HCC). It was first described in 1985 as a gastric tumor with unique clinicopathological features of tumor cells resembling hepatocytes with eosinophilic cytoplasm, large centrally located nuclei, and alpha-fetoprotein (AFP) expression. Since then, it has been identified as a primary tumor in other sites, with the stomach accounting for the majority of reported cases (63%), followed by the ovaries, gallbladder, pancreas, and uterus [1]. Hepatoid adenocarcinoma of the lung (HAL) represents less than 5% of HAC cases and is characterized by histologic resemblance to hepatocytes and, variably, AFP expression. In 1990, Ishikura and colleagues first reviewed seven cases of AFP-producing lung cancer and proposed diagnostic criteria for HAL. The criteria adopted for the diagnosis of hepatoid adenocarcinoma are: (1) an admixture of tubular or papillary adenocarcinoma with AFP-positive tumor tissue forming sheet-like or trabecular patterns, and (2) tumor cells in these areas characterized by abundant eosinophilic cytoplasm and centrally located nuclei, features reminiscent of hepatocellular carcinoma [2]. In subsequent reports, although AFP expression on histopathology and/or elevated serum AFP levels were described in most cases of HAL, not all HALs were found to produce AFP in clinical practice [3]. Based on these observations, Haninger et al redefined the criteria for HAL diagnosis cited above as follows: (1) HAC may present either as a purely hepatoid tumor or alongside other histologic types, including acinar or papillary adenocarcinoma, signet-ring cell features, or neuroendocrine carcinoma, and (2) AFP expression is optional when other markers consistent with hepatic differentiation are identified [4]. Although HAL remains exceedingly rare, an increasing number of cases have been reported in recent years, likely due to improved histopathological recognition and broader use of immunohistochemical markers for hepatic differentiation [5,6]. Despite these advances, HAL continues to pose diagnostic challenges, especially when it presents with atypical features or at uncommon metastatic sites.

## Case presentation

The patient is a 64-year-old male with a medical history of hypertension, dyslipidemia, and type 2 diabetes mellitus, managed with valsartan/hydrochlorothiazide, atorvastatin, and metformin. He had a significant smoking history of 50 pack-years. Family history was non-contributory, and he had no other relevant social or occupational exposures.

**Figure 1:**
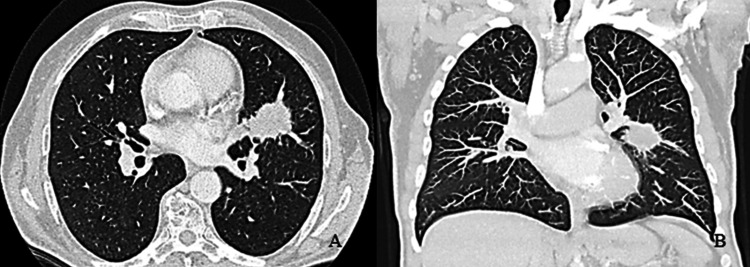
(A) Axial and (B) coronal CT scan (lung window) showing the left upper lobe lesion

He was evaluated for a left upper lobe lobulated solid lesion. Physical examination revealed clubbing and peripheral edema, consistent with hypertrophic pulmonary osteoarthropathy. His blood work, including serum AFP, was within normal limits. Two months prior, the patient presented with a solid, palpable, painful mass in the right submandibular region with an intraoral projection. Head and neck imaging with computed tomography (CT) and magnetic resonance imaging (MRI) demonstrated a 30 × 16 mm soft-tissue mass in the medial aspect of the right mandible, abutting the mandible and causing cortical rim erosion. A chest CT scan revealed a 35x33 mm solid lesion in the left upper lobe with no pathological lymph nodes (Figure 1).

**Figure 2:**
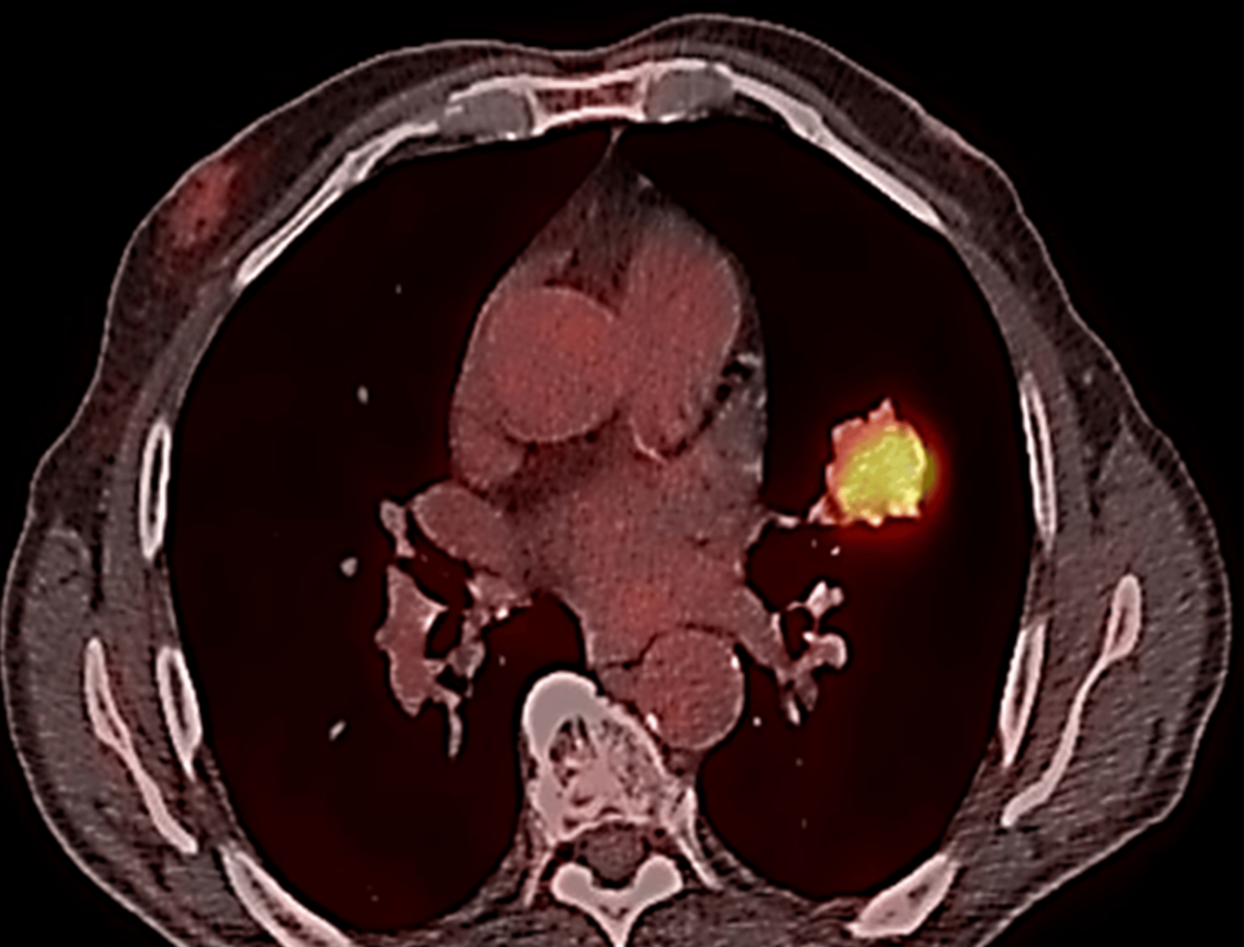
PET-CT scan showing increased uptake of FDG from the lesion (SUVmax: 8.8)

Positron emission tomography using fluorodeoxyglucose (FDG-PET) detected a hypermetabolic lesion anterior to the mandible, in contact with the inferior aspect of the masseter muscle, not infiltrating the submandibular gland, and an abnormal uptake in the left upper lobe tumor (maximum standard uptake value, SUVmax= 8.8) (Figure 2).

**Figure 3:**
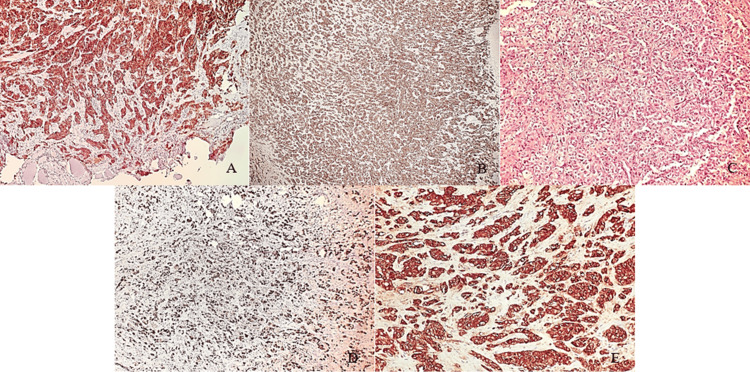
Immunohistochemical and histopathological analysis. (A) Cytokeratin 7 (CK7) immunostaining shows strong and diffuse cytoplasmic positivity. (B) AE1/AE3 immunostaining demonstrates diffuse cytoplasmic positivity, further confirming epithelial differentiation. (C) Hematoxylin and eosin (H&E) staining reveals sheets of atypical cells characterized by prominent nucleoli and a high nuclear-to-cytoplasmic ratio. (D) Hepatocyte-specific antigen (HepPar-1) staining shows diffuse nuclear positivity, supporting hepatoid differentiation. (E) Epithelial membrane antigen (EMA) staining shows localized brown cytoplasmic positivity.

He underwent fine needle aspiration (FNA) of the submandibular lesion and an intraoral tissue sampling, both revealing high grade salivary gland carcinoma, not otherwise specified (NOS). Bronchoscopy of the lung mass revealed adenocarcinoma with no malignant findings on endobronchial ultrasonography-transbronchial needle biopsy (EBUS-TBNB) of lymph node stations 4L and 7. Brain CT and MRI imaging showed no malignant findings, and abdominal MRI was negative for liver parenchymal lesions. The pathology report of the subsequent right-sided partial gnathectomy, which resulted in complete excision of the metastatic mass, indicated morphological and immunohistochemical findings suggestive of hepatoid carcinoma. In July 2024, he underwent an open left upper lobe lobectomy with mediastinal lymph node dissection. The pathological review revealed a grayish tumor measuring 4.5×4×3.5 cm, while immunohistochemistry staining demonstrated: hepatocyte (+), cytokeratin 7 (CK7) (+), CK18 (+), Synaptophysin (+), Chromografin (+), CDX2 (-), TTF-1 (+), Napsin (+), AE1/AE3 (+), CK20 (-), CK5/6 (-), CK14 (-), CD56 (+), p40 (-), Estrogen (-), Androgen (-), Vimentin (-), Calponin(-), p63 (-), DOG-1 (-), SOX-10 (-), LCA (-), SMA (-), S-100 (-), C-erb-2 (-), p53 (-) (Figure 3). Based on the morphological and immunochemical findings, the diagnosis of primary hepatoid adenocarcinoma with neuroendocrine differentiation and hilar lymph node invasion was made (T2bN1M1b, Stage IVA as per the 8th edition of the TNM staging system for lung cancer) [7]. Within one month after surgery, while the patient was receiving a dual platinum-based chemotherapy regimen consisting of cisplatin and pemetrexed administered according to standard protocols, increasing pelvic and back pain prompted further imaging. MRI scans identified bony metastases in the right sciatica and lumbar vertebrae. The patient underwent radiation therapy for the bony metastases; however, he showed no signs of remission and succumbed to his illness 4 months post-surgery, 6 months after diagnosis was established.

## Discussion

Hepatoid adenocarcinoma of the lung is a rare and aggressive subtype of non-small cell lung cancer (NSCLC), defined by its morphological and immunophenotypic resemblance to HCC despite a primary pulmonary origin. The current consensus in the literature indicates that HAL predominantly affects middle-aged individuals with a heavy history of tobacco use and exhibits a strong male predominance [8,9]. This sex disparity may be partially explained by the high prevalence of tobacco use among male patients. Interestingly, female patients appear to have a more favorable prognosis, though the mechanisms underlying this potential sex-based difference remain poorly understood, and the insufficient number of reported cases prevents drawing safe conclusions [6].

Patients with HAL have a non-specific clinical presentation with symptoms including cough, chest pain, dyspnea, hemoptysis, and systemic symptoms such as anorexia, fatigue, and weight loss. Anatomically, HAL tends to involve the upper lobes and shows a predilection for the right lung [4,6]. Tumors are typically large at the time of diagnosis, and both lymph node involvement and distant metastases are common at initial presentation [8]. Although metastases to uncommon sites, namely in the tonsil and gingiva, have been reported in the literature, our case exhibits a particularly unusual presentation [11,12]. Based on available data, this appears to be the first case describing HAL with mandibular metastasis, which was also the initial clinical presentation of the disease.

Diagnosis of HAL is challenging due to its overlapping clinical and radiological features with other malignancies. Radiologically, HAL may present as a large mass with or without cavitation or necrosis [13], and is often indistinct from other aggressive lung tumors, while serum AFP levels are often elevated. Thus, definitive diagnosis is based on histopathological examination and immunohistochemical profiling. Macroscopically, the tumors are typically large, well-circumscribed, gray in color, and often necrotic [14]. On pathology, the tumors demonstrate areas of hepatoid differentiation with large, polygonal, eosinophilic or hyaline cells with a mixture of regions with glandular differentiation, poorly differentiated adenocarcinoma, or neuroendocrine features [4,15]. HAL often expresses AFP, HepPar-1, CK7, CK8/18, TTF-1 (cytoplasmic), though expression patterns vary among reported cases [4,16,17].

Differential diagnosis of HAL involves other types of primary pulmonary adenocarcinoma, pulmonary metastases of HCC or of hepatoid adenocarcinoma originating elsewhere, and germ cell and neuroendocrine tumors [10,14]. AFP is a glycoprotein produced at very low levels in adults and can serve as a significant biomarker in various clinical contexts. AFP production is commonly observed in HAC and can be used as a tumor marker, being a sensitive indicator to monitor therapeutic response, relapse, and aggravation, often preceding any radiologic evidence [13]. However, its absence does not exclude the diagnosis, underscoring the importance of morphologic and immunohistochemical evaluation [4]. While Papatsimpas et al. observed that patients presenting with normal AFP concentrations often show extended overall survival, other studies have failed to highlight consistent prognostic value in AFP expression [6,11,18]. Serum AFP levels have been shown to decline postoperatively after resection of primary tumors, likely reflecting reduced tumor burden and explaining discrepancies between pretreatment tissue expression and post-treatment serum normalization [13,19]. In our patient, while the serum AFP was within normal range prior to treatment of the primary tumor, surgical removal of a solitary metastasis had preceded the sampling. Currently, treatment options for HAL follow the management protocols used for treating classical NSCLC. Surgical resection is recommended when complete excision is feasible, whereas advanced HAL is managed with chemotherapy, commonly platinum-based, and radiotherapy. In recent years, while targeted therapies and immune checkpoint inhibitors show promise in selected cases, treatment is largely experimental, and no standardized protocol exists [6,15,20]. Despite these advances, outcomes in patients with HAL remain extremely poor. Both vascular invasion [1] and advanced stage at diagnosis are unfavorable prognostic factors, with disease stage representing the strongest predictor of overall survival [21]. With a reported median overall survival of 14 months, HAL follows a more aggressive clinical behavior than other NSCLCs [10].

## Conclusions

In conclusion, HAL is a rare primary pulmonary malignancy with an extremely poor prognosis and no standardized treatment. We presented our experience underscoring key clinical features of HAL, along with comprehensive immunohistochemical profiling and an overview of its management. To our knowledge, this is the first reported instance of HAL with mandibular metastasis as the initial presentation. These findings highlight the importance of considering HAL in the differential diagnosis of poorly differentiated lung tumors, especially in patients with unusual metastatic patterns. Given the aggressive nature of the disease, early recognition and appropriate management are crucial to improving outcomes.
